# Access to Chagas disease treatment in the United States after the regulatory approval of benznidazole

**DOI:** 10.1371/journal.pntd.0008398

**Published:** 2020-06-22

**Authors:** Kota Yoshioka, Jennifer Manne-Goehler, James H. Maguire, Michael R. Reich

**Affiliations:** 1 Doctor of Public Health Program, Harvard T.H. Chan School of Public Health, Boston, Massachusetts, United States of America; 2 Division of Infectious Diseases, Massachusetts General Hospital, Boston, Massachusetts, United States of America; 3 Division of Infectious Disease, Brigham and Women’s Hospital, Harvard Medical School, Boston, Massachusetts, United States of America; 4 Department of Global Health and Population, Harvard T.H. Chan School of Public Health, Boston, Massachusetts, United States of America; Universidade Federal de Minas Gerais, BRAZIL

## Abstract

Approximately 300,000 persons in the United States (US) are infected with *Trypanosoma cruzi*, the protozoan that causes Chagas disease, but less than 1% are estimated to have received antiparasitic treatment. Benznidazole was approved by the US Food and Drug Administration (FDA) for treatment of *T*. *cruzi* infection in 2017 and commercialized in May 2018. This paper analyzes factors that affect access to benznidazole following commercialization and suggests directions for future actions to expand access. We applied an access framework to identify barriers, facilitators, and key actors that influence the ability of people with Chagas disease to receive appropriate treatment with benznidazole. Data were collected from the published literature, key informants, and commercial databases. We found that the mean number of persons who obtained benznidazole increased from just under 5 when distributed by the CDC to 13 per month after the commercial launch (from May 2018 to February 2019). Nine key barriers to access were identified: lack of multi-sector coordination, failure of health care providers to use a specific order form, lack of an emergency delivery system, high medical costs for uninsured patients, narrow indications for use of benznidazole, lack of treatment guidelines, limited number of qualified treaters, difficulties for patients to make medical appointments, and inadequate evaluation by providers to determine eligibility for treatment. Our analysis shows that access to benznidazole is still limited after FDA approval. We suggest six areas for strategic action for the pharmaceutical company that markets benznidazole and its allied private foundation to expand access to benznidazole in the US. In addition, we recommend expanding the existing researcher-clinician network by including government agencies, companies and others. This paper’s approach could be applied to access programs for benznidazole in other countries or for other health products that target neglected populations throughout the world.

## Author summary

Less than 1% of persons in the United States with *Trypanosoma cruzi* infection, the cause of Chagas disease, have received antiparasitic treatment. In this paper, we examine the status of access to benznidazole in the United States following its approval by the FDA for treatment of Chagas disease treatment in 2017 and its commercialization in 2018. We found that the average number of patients who received benznidazole from the manufacturer increased after commercialization from just under 5 (when distributed by the CDC) to 13 patients (when sold in commercial channels) per month. We identified various barriers to access to treatment such as problems in the drug ordering process, low demand from health care providers, inadequate clinical assessment of patient eligibility for treatment, obstacles for patients to make medical appointments or to pay medical costs, and weak coordination among key actors in the health system. To overcome these access barriers, we propose strategic actions that can be taken by the pharmaceutical producer in collaboration with other key actors. Our work contributes to expanding access to Chagas disease treatment in the United States, and our approach could be applied to efforts to expand access to health products for other neglected diseases.

## Introduction

In the United States (US), approximately 300,000 persons, nearly all immigrants from Latin America, are infected with *Trypanosoma cruzi*, the protozoan cause of Chagas disease (CD) [1,2]. In a recent study, 1.24% of Latin American-born residents of Los Angeles County attending health fairs were infected with *T*. *cruzi* [3]. CD-related annual healthcare costs are estimated to be $118 million USD in the US, representing nearly 20% of estimated global costs [4]. Screening for CD in the early stages of disease and provision of appropriate treatment and disease management could reduce the substantial public health burden for this neglected tropical disease in the US.

Two antitrypanosomal agents, benznidazole and nifurtimox, have activity against *T*. *cruzi* and offer benefit for some CD patients. Benznidazole treatment is indicated for persons with acute, congenital or reactivated infections, infected children and women of childbearing age to prevent transplacental transmission; although it is still unknown whether treatment can prevent development of heart disease, it is usually offered to infected adults under the age of 50 without well-established Chagas cardiomyopathy [5–7]. The safety profile of benznidazole is far from optimal. Frequent adverse reactions are relatively mild, including skin reactions, gastrointestinal complaints, and neurological symptoms, while 3% of cases develop severe reactions, such as peripheral neuropathy, toxic hepatitis, Lyell syndrome, anaphylactic reaction, and Stevens-Johnson syndrome [8]. The adverse reactions can lead to treatment discontinuity, and patients under benznidazole treatment need careful medical follow-up.

In the US, benznidazole had been available since 2011 under investigational protocols from the Center of Disease Control and Prevention (CDC) [9]. However, a study in 2015 showed that less than 1% of those estimated to be infected in the US had been identified and few had received treatment [10]. The same study found that access to CD treatment was severely limited because of health system barriers, including scarce opportunities for diagnosis and follow-up, inadequate financing for medical care, lack of drug approval by the US Food and Drug Administration (FDA), and limited awareness and training among health care providers [10]. In short, CD treatment was not accessible to the vast majority of *T*. *cruzi* infected persons in the US.

A milestone was reached on August 29, 2017, when the FDA approved benznidazole for use in children aged 2 to 12 years old [11]; in addition the drug can be prescribed legally to patients of other ages, a common practice called “off-label” use. The FDA granted a neglected tropical disease priority review voucher (PRV) to the drug manufacturer, Chemo Research (renamed Insud Pharma). The PRV program is meant to incentivize drug development for neglected diseases. A PRV can be used by the manufacturer to accelerate FDA review for a different drug or can be sold to other companies [12]. Insud Pharma publicly announced its commitment to invest a substantial part of the financial benefits from the sale of the PRV to enhance access to CD treatment and improve patient health in other disease areas [13].

FDA approval followed by commercialization changed the supply chain of benznidazole in the US in significant ways. During the pre-license period, benznidazole was available under FDA Investigational New Drug (IND) mechanisms, which required an IRB-approved protocol with appropriate consenting and definition of treatment eligibility. CDC purchased benznidazole from the manufacturer and established such a protocol under FDA IND mechanisms, making it available from October 2011. Following FDA approval, on May 14, 2018, CDC discontinued its benznidazole program, and Exeltis USA began to market Insud Pharma’s Benznidazole Tablets. Exeltis USA is a private company owned by Insud Pharma and sells branded medicines in the US in the areas of women’s health and dermatology. Benznidazole became available through commercial channels, which was expected to make obtaining benznidazole less complex for physicians and to expand access to the medicine for infected individuals [10].

It is well known from experience, however, that FDA approval and commercialization of a medicine can still confront problems in expanding access for patients. For example, miltefosine, a life-saving drug for leishmaniasis, was approved by the FDA in 2014 and a PRV was granted to the manufacturer, but access to the medicine remains limited [14]. This paper therefore examines factors influencing access to benznidazole in the post-license period of commercialization and proposes actions that the manufacturer and others can take to expand access to benznidazole for infected individuals in the United States.

## Methods

### Conceptual framework

This paper uses an access framework, developed by Frost and Reich [15], to analyze access to benznidazole in the United States and to recommend strategic actions. According to Frost and Reich [15], “a deeper understanding of the facilitators, barriers and key actors […] is necessary” (p. 18) to “develop strategies for guiding the [health] technology to the end-user” (p. 34). Access is defined as “an end-user’s ability to consistently obtain and appropriately use good quality health technologies when they are needed” (p. 219). Access can be shaped by four activity streams: architecture (the organizational structure and relationships); availability (the supply components); affordability (the cost issues for various players); and adoption (demand factors and acceptance). This framework has been used, for instance, to identify access barriers to medicines for leishmaniasis in Africa [16] and to inform development of a new accessible diagnostic for soil-transmitted helminths [17].

This access framework was chosen for several reasons. First, the framework focuses on access as a single outcome. While multiple factors interact within a health system, the framework offers a simplified model to frame issues around access. Second, the framework is comprehensive enough to capture the roles of multiple stakeholders ranging from international organizations to end-users. Third, the framework restricts its scope to health technologies, such as medicines, diagnostics and vaccines, which gives clarity to strategies that this paper seeks to propose.

**Fig 1:**
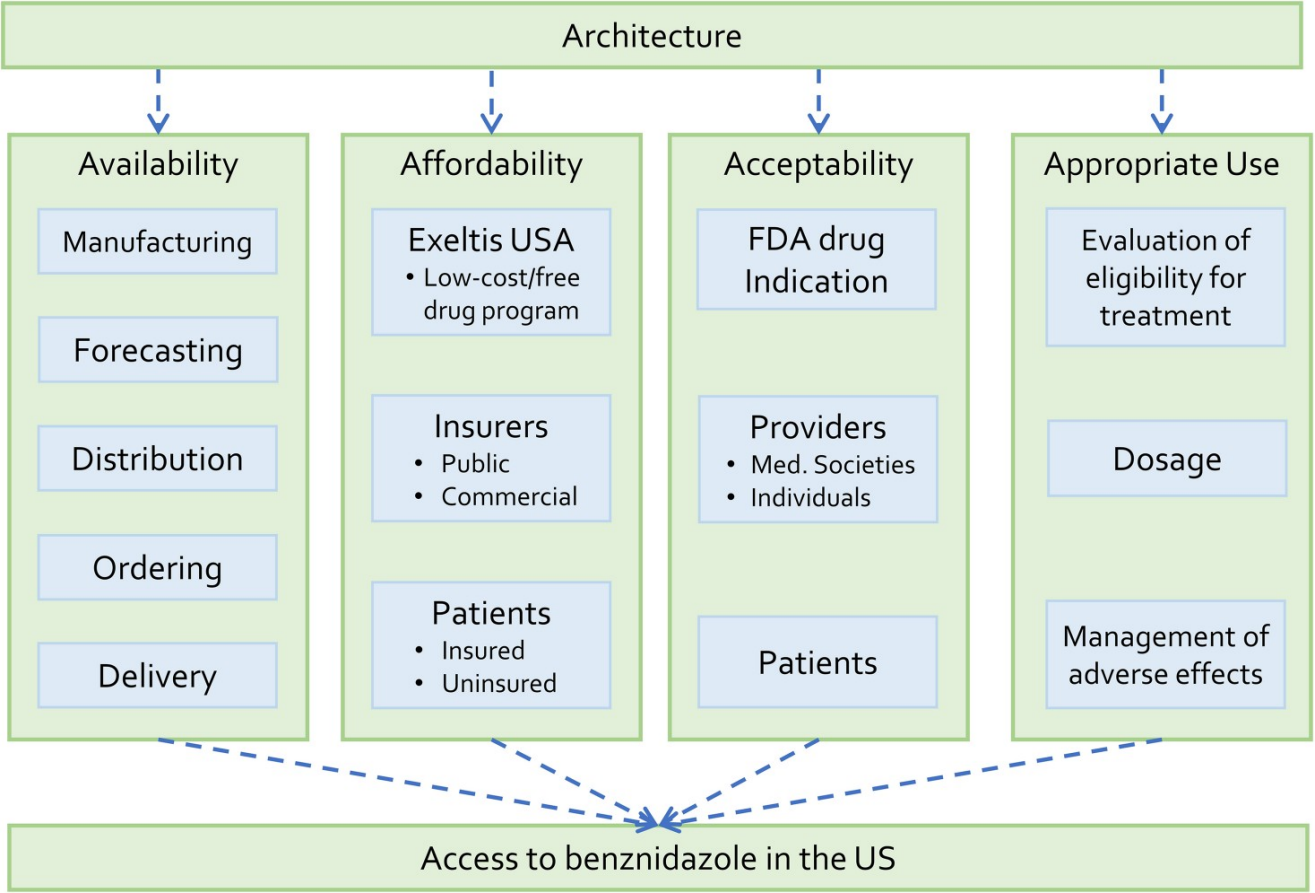
Access framework for benznidazole in the United States. Adapted from Frost and Reich [15].

We adapted the access framework [15] to the case of benznidazole in the US (Fig 1). The authors of the original framework recommend modification to take into account features of the specific technology and national context. Adaptation was necessary in part because the original framework focuses on low- and middle-income countries. We decided on four key modifications. First, in the stream of availability, procurement was replaced by ordering. This adaptation reflects the change in distribution of benznidazole in the US from the CDC program of compassionate use (based on procurement) to a company-based system based on health care providers who prescribe medications for individual patients. Second, in the stream of affordability, the pharmaceutical company (Exeltis USA) and insurers are included as payers. The original framework considers governments, non-governmental agencies and end-users as payers. In the US, drug costs are typically shared among governmental and commercial insurance schemes, patients, and a pharmaceutical company’s patient assistance program. Third, in the stream of adoption, the analysis of global adoption was removed. Our assessment of Chagas disease treatment in the US suggests that global adoption factors (such as the World Health Organization’s policies) have not had major influence on access to benznidazole in the US context. Fourth, the stream of adoption was divided into “acceptability” and “appropriate use.” The original framework considers appropriate use as part of patient adoption. For benznidazole, appropriate use is determined not only by patient behavior, but also by physician decisions and practices.

In our adapted framework, access to benznidazole refers to CD patients’ ability to obtain and appropriately use benznidazole when they require antiparasitic treatment. Throughout this paper, CD patients mean persons whose *T*. *cruzi* infection has been already diagnosed and who could be considered for treatment. We provide adapted definitions of the four activity streams in the Results section.

### Data collection and analysis

Data were collected from multiple sources, including: 1) published literature that identified access barriers in the pre-license period; 2) informal interviews, meetings, and e-mail exchanges with key informants who had expertise and experience in addressing CD treatment in the US, including nine health care providers, five Exeltis USA employees, four researchers, and one government officer; and 3) internal information provided by Exeltis USA and Fundación Mundo Sano (FMS). FMS is a non-profit private foundation established by Insud Pharma, and aims to reduce the impact of neglected diseases worldwide. Data were collected between July 2018 to March 2019. Key informants were identified using snowball sampling methods. We began our interviews with several key informants who were members of the Chagas Consortium (see below) and Exeltis USA, then asked each interviewee to identify other persons who had experience or were knowledgeable about CD treatment in the US. We prepared different lists of interview topics, according to the interviewees’ background and roles. For example, we asked about logistics and pricing of benznidazole to Exeltis USA employees, while we explored provider and patient perspectives with physicians who had attended CD patients.

Data were analyzed in two ways. First, we compared the trend in access to benznidazole before and after commercial launch on May 14, 2018, using data on drug dispensing. To quantify the level of access to benznidazole, we used the number of patients to whom benznidazole was dispensed per month. This indicator reflects the ability of CD patients to obtain benznidazole from Exeltis USA, but it does not necessarily reflect demand for the drug or patients’ ability to use benznidazole appropriately. In addition, we compared the age distribution of CD patients who obtained benznidazole before and after commercialization, which has implications for appropriate use.

Second, we analyzed qualitative data to identify barriers, facilitators and actors that shape access to benznidazole after the commercial launch. To identify barriers (conditions limiting access) and facilitators (conditions promoting access), we deductively compared the access framework and the collected data. A barrier was identified when the access framework suggested that a certain condition was necessary to create access, but the collected data showed that this condition was not satisfied. Likewise, a facilitator was identified when a necessary condition deduced from the access framework was shown to be satisfied by the collected data. In addition, the authors examined potential processes through which the barrier or facilitator would affect access to benznidazole in the US. Actors were individuals or organizations that played or were expected to play a role in relation to the barriers or facilitators. In this analysis, we reported all the barriers, facilitators and actors that were identified and classified them within the framework. This classification reflected an agreement of the authors.

## Results

### Trend of access to benznidazole after commercialization

The monthly average of CD patients who obtained benznidazole increased from just under 5 persons during the period in which CDC provided benznidazole [16], to 13 on average in the nine months following commercialization on May 14, 2018. Despite the observed increase, the overall number of treated persons was still small even after commercialization, compared to the large number of estimated people infected with *T*. *cruzi* in the US (although not every infected person needs benznidazole). While the proportion of patients between 2–12 years old prescribed benznidazole increased after the commercial launch, more than 90% of persons treated were adults aged over 19 years in both periods (Table 1). Despite the FDA’s indication for pediatric use, benznidazole has been largely used to treat adult patients in the US.

**Table 1:** Age distribution of Chagas disease patients to whom benznidazole was dispensed before and after commercialization in May 2018.

	Oct 2011- May 2018		May 2018- Feb 2019	
Age group (yrs)	No	%	No	%
<2	1	0.3	0	0.0
2–12	2	0.5	5	3.9
13–18	29	7.9	2	1.6
19–50	236	64.7	80	62.0
>50	97	26.6	42	32.6
**Total**	**365**	**100**	**129**	**100**

Source: Herwaldt et al. [9], Exeltis USA

### Key barriers, facilitators and actors shaping access to benznidazole

Key factors that affect access to benznidazole are summarized in Table 2. The table presents the barriers and facilitators to access, and actions to address challenges to access associated with each of the streams that influence access (architecture, availability, affordability, and appropriate use). These key factors are briefly discussed next.

**Table 2:** Key barriers and facilitators shaping access to benznidazole (BNZ) in the United States.

Stream	Barriers	Facilitators	Actions to Address Challenges
Architecture	- Lack of a national level network for multi-sector coordination.	- Existence of two networks (Chagas Consortium and Texas Chagas Task Force).	- Create a national platform as a mechanism for multi-sector coordination.
Availability	- Providers’ failure to send a specific order form to a specialty pharmacy when they prescribe BNZ. - Lack of an express delivery system for emergency situations.	- BNZ production and importation controlled by the single corporate group. - Forecasting leading to adequate supply of benznidazole, compared to the current demand level. - A specialty pharmacy providing logistic assistance for domestic delivery of BNZ.	- Increase provider awareness about the order form. - Develop an emergency BNZ delivery system.
Affordability	- Costs of medical services for uninsured patients.	- Exeltis USA’s low-cost/free drug program. - Some of the major public and private insurers covering BNZ.	- Establish financial sustainability of the Exeltis USA’s low-cost/free drug program. - Expand public and private insurance coverage for BNZ.
Acceptability	- Narrow indications for BNZ approved by the FDA. - Lack of a clinical practice guideline for CD treatment. - Limited number of physicians offering CD treatment. - Obstacles for patients to make medical appointments.	- FDA approval of BNZ. - Existence of treatment centers.	- Expand the FDA-approved indication of BNZ. - Establish a clinical practice guideline for CD treatment. - Organize a physician network. - Organize a patient peer support network.
Appropriate use	- Providers not confirming diagnosis or inadequately evaluating patient eligibility for CD treatment.	- Clinical guidance available from CDC	- Educate prescribers on how to confirm diagnosis and evaluate patient eligibility for BNZ treatment. - Understand treatment practices (dosage and management of adverse effects).

BNZ: Benznidazole, CD: Chagas disease

Source: Authors.

#### Architecture

Architecture refers to the network of organizations that steer and connect activities to expand access to benznidazole. Key actors need to be organized into effective relationships and partnerships, which can require establishing an effective governance structure among partners, transparency in decision-making, and program accountability [15].

Two networks are found active in the US. First, the Chagas Consortium is an informal group of approximately 20 researchers and clinicians committed to improving the availability of CD diagnosis and treatment. Its members frequently exchange information but there is no formal structure of leadership and membership. Second, the Texas Chagas Task Force was created in 2015 with CDC funding in order to raise awareness of CD and improve screening and treatment in that state [18]. This network has over 100 members, including researchers, public health agencies, entomologists, veterinarians, and health care providers in Texas. The group has one assigned coordinator who organizes quarterly online meetings.

Despite these two networks, there is no structured form of collaboration at the national level for Chagas disease in the US. This situation could be improved to coordinate interests not only among individual researchers and clinicians but also among government agencies, non-governmental organizations such as blood banks, and companies including Exeltis USA.

#### Availability

Availability refers to the degree to which benznidazole reliably reaches CD patients. Benznidazole needs to be manufactured with adequate demand forecasting, distributed from the manufacturer to pharmacies, prescribed and ordered by health care providers, and delivered to patients.

Production of benznidazole is controlled by Insud Pharma. Benznidazole for the US market is manufactured by Insud Pharma’s two plants in Spain: Quimica Sintetica, which produces active pharmaceutical ingredients, and Liconsa CMO, which fabricates Benznidazole Tablets. From Spain, benznidazole is imported and stored at Foundation Care in St. Louis, Missouri, a specialty pharmacy contracted by Exeltis USA. Foundation Care provides Exeltis USA with warehousing services, transactions of prescriptions, and domestic shipment of benznidazole. While Exeltis USA had imported benznidazole only once as of March 2019, the supply of benznidazole to the US market is expected to be reliable.

For demand forecasting, Exeltis USA estimated 200 patients to be treated with benznidazole per year. To date, Exeltis USA has imported 2,000 bottles of benznidazole, which are sufficient to treat approximately 1,000 patients. As of February 2019, the average number of patients who obtained benznidazole was 13 per month, suggesting that the annual number of patients might not reach the estimated number of 200 in 2019. Exeltis USA is oversupplying benznidazole, but this is not an access barrier since the surplus will not limit access.

To prescribe and order benznidazole, health care providers need to fax a two-page order form to Foundation Care. The form is available on Exeltis USA’s Benznidazole Tablet website (https://www.benznidazoletablets.com). Submission of this order form is required because Foundation Care needs to collect data to determine if a patient is eligible for co-pay assistance or product at no charge. Some cases have been observed in which providers prescribed benznidazole without submitting the order form and thus failed to provide drugs to patients. No data are available about how many prescriptions are currently rejected at retail pharmacies in this way, but the failure to send the order form can be a critical barrier.

**Fig 2:**
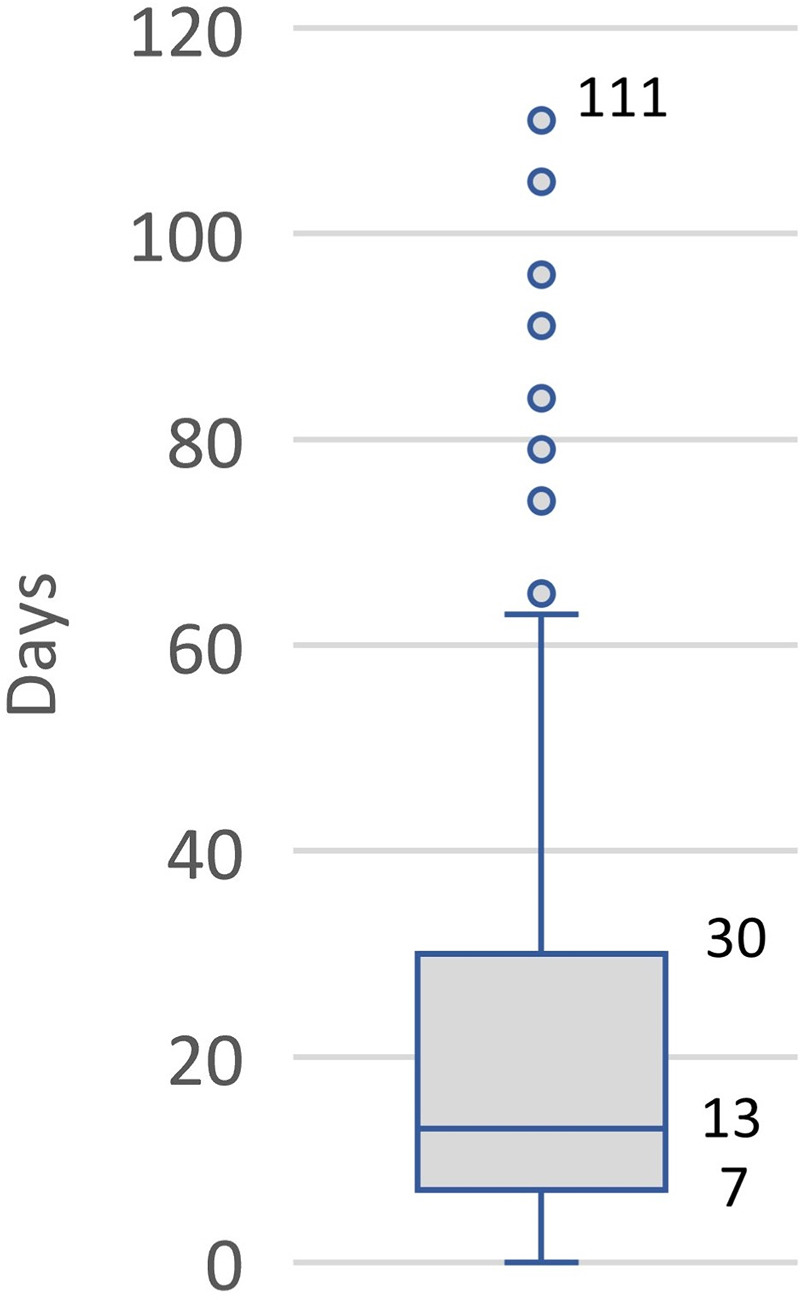
Box plot showing durations in days between the dates when benznidazole is prescribed and dispensed for 129 patients, May 2018 to February 2019. Source: Exeltis USA.

Delivery of benznidazole has experienced some significant delays. In 75% of cases, it took more than seven days for Foundation Care to dispense benznidazole after a provider submitted a prescription (Fig 2). Some outlier cases took more than 60 days, due to various reasons, including: 1) the pharmacy team at Foundation Care sometimes had difficulty contacting the patient or the prescribing physician to clarify information in the order forms, 2) insurance companies required preauthorization, and 3) patients delayed making an out-of-pocket payment. The reasons for and consequences of this long period of time between prescription and dispensing are not clear and additional study may be required.

Emergency situations, such as acute transplant-transmitted infections or reactivation disease in the setting of HIV, do not occur frequently (probably less than 10 cases annually) but benznidazole or nifurtimox needs to be delivered as soon as possible in those situations. Exeltis USA is responsible for this emergency delivery service but has not yet established a system for it as of March 2020. The major setback is the time that it takes for Foundation Care to process the prescription, that is, to receive the prescription, assess coverage, request a prior authorization (if necessary), enroll in a patient assistant program (if applicable), and dispatch the medicine.

#### Affordability

Affordability refers to the degree to which CD patients can pay for benznidazole and related services. In the US, the main payers involve Exeltis USA, public and commercial insurers, and CD patients. Appropriate cost-sharing is crucial for patients to get affordable CD treatment.

As of March 2019, the price of benznidazole was $300 USD for a bottle of 100 mg tablets and $250 USD for 12.5 mg (100 tablets per bottle). The doses needed for treatment depend on patient body weight, but Exeltis USA estimates that the average drug cost per 60-day treatment course would be $600 USD.

To improve benznidazole affordability, Exeltis USA launched a subsidy program called FastAccess. This program limits patients’ co-payments to $60 USD when their health insurance covers benznidazole and provides free drugs if patients have no insurance or if their insurance does not cover benznidazole. The subsidy is not applied to patients covered by government-funded insurance programs because of the anti-kickback law. Medicaid users’ co-payments have been usually less than $10 USD per treatment course, with some exceptions in which patients were required to pay $40–90 USD per 30-day treatment. As of the second week in March 2019, the total amount paid for benznidazole has been shared among FastAccess (43%), commercial insurance companies (34%), government insurance programs including Medicaid, Medicare, and Tricare (14%), and patients (8%). The FastAccess program and expanded insurance coverage for benznidazole have been key facilitators.

The FastAccess program is financed by FMS. The program’s financial sustainability should be evaluated since its discontinuation could be a critical barrier to access in the future. Financial reliance on the FastAccess program can be decreased by increasing insurance coverage of benznidazole. Exeltis USA needs to advance negotiations with insurers that have not yet covered benznidazole.

Out-of-pocket expenses for related medical services can be problematic particularly for uninsured patients, who can be required to pay for all the services including serological tests, several physician visits and electrocardiogram. Some uninsured patients may be eligible for private insurance offering affordable plans for people living with low income. However, the issue of unaffordable health care stems from the complex nature of the US health system, which is beyond the scope of this paper.

#### Acceptability

Acceptability refers to the degree to which benznidazole is accepted and demanded by various components of the health system. In the US, this includes the FDA, medical societies, health care providers, and patients.

FDA approval of benznidazole is a key facilitator for acceptability, but the indication for use in children aged 2 to 12 years old has two major limitations. First, Exeltis USA is not legally allowed to promote off-label use of benznidazole for adult patients, who constitute the greatest number of persons in the US who would benefit from the drug. Second, the narrow indication may directly limit physician demand. Despite CDC’s and experts’ recommendations to treat certain groups of adult patients [19,20], robust scientific evidence is still lacking to demonstrate health benefits from benznidazole treatment of most adult patients, other than women of childbearing age to prevent transplacental infection [7]. While the drug is currently used mostly for treatment of adult patients (Table 1), this practice can be controversial and physicians may avoid making difficult decisions without a clear message from the FDA.

Few US medical societies have guidelines for treating Chagas disease. A review article published in JAMA [6] and the CDC website are common sources of information for physicians to make treatment decisions, but these two sources have limited information on management of adverse effects. Algorithms to evaluate CD in pregnant women and infants in the US were published recently in a review article [21]. Development and endorsement of a clinical practice guideline [22] could help in establishing widely accepted norms of practice for CD treatment in the US.

A small number of physicians has requested benznidazole since its commercial launch in May 2018. As of December 2018, 70 physicians had ordered benznidazole, including only 11 physicians who treated more than one CD patient. As of February 2019, 22.5% of CD treatment with benznidazole was concentrated in three treatment centers: 1) Boston Medical Center, Boston, Massachusetts; 2) Center of Excellence for Chagas Disease, Sylmar, California; and 3) Latin American Society of Chagas, Bristow, Virginia. Emergence of additional local treatment centers could facilitate access to benznidazole; however, it is challenging for most CD patients living in the US to find physicians who can provide adequate care. Poor physician awareness and knowledge about CD have been repeatedly emphasized as problems [10,23–26]. Organizing a physician network could help increase the number of providers offering treatment and also improve physician acceptance of benznidazole [18].

Patients’ demand for benznidazole should be sustained while they seek care and complete a 60-day treatment course. According to the Boston Medical Center’s experiences, patients often struggle or even fail to make several medical appointments for treatment due to difficulties to accommodate job requirements or childcare duties, high transportation and opportunity costs, or insurance requirements. Similar problems are reported among patients in Los Angles [27]. Support for patients is critical to keep patients engaged and encouraged during the process of seeking and receiving treatment. One potential solution is “patient peer support” (provision of help and support from treated patients to others) that has been used to improve cancer care [28].

#### Appropriate use

Appropriate use refers to the degree to which benznidazole is effectively and safely used. Appropriate use requires health care providers to confirm the diagnosis and determine eligibility for treatment by evaluating stage of disease, age, and pregnancy status, among others [6]. It also requires determination of appropriate dose and duration, as well as frequent medical checkups to manage adverse effects.

Before benznidazole became commercially available in May 2018, the CDC worked with providers to confirm diagnosis and assess patient eligibility for treatment. Currently, the CDC continues to provide teleconsultation services regarding CD treatment [9], however, no institution is playing a gatekeeping role and providers can obtain benznidazole on their own initiative. No information is available to understand how often Exeltis USA’s benznidazole was dispensed for treatment of a patient whose infection was not confirmed. Some CD experts are concerned about the potential misuse of benznidazole. Indeed, our study found some cases in which a physician prescribed benznidazole without confirming the diagnosis or prescribed it inappropriately, for example when benznidazole was prescribed to an 88-year-old patient (the drug is not generally recommended for patients over 50 years old [6]). Inadequate evaluation of patient eligibility for treatment could be a key barrier to appropriate use. Information on how to confirm diagnosis and evaluate patient eligibility could be shared among health care providers who prescribe benznidazole in order to improve treatment practices.

Only limited information is available to understand whether physicians select correct dosage and manage adverse effects appropriately. Some cases of potential overuse were found in Exeltis USA’s data, in which physicians ordered two or three times more benznidazole tablets than are recommended for an adult patient. However, dosage cannot be calculated by the company because patient weights are not reported. Post-marketing safety surveillance of benznidazole is performed via the FDA Adverse Event Reporting System, but it does not provide information to assess whether physicians are providing adequate medical supervision. More information is necessary to understand how providers currently manage benznidazole treatment.

## Discussion

Frost and Reich [15] emphasize that regulatory approval of a medicine is a midway success point in a complicated process of improving access to health technologies in developing countries. Our study confirmed a similar pattern with the case of benznidazole in the United States. We found that the use of benznidazole increased after commercialization but that many challenges still need to be addressed to significantly expand access to this medicine (Table 2).

There are multiple potential reasons for the observed increase in the use of benznidazole after commercialization, including: 1) simplified paperwork for physicians for ordering after commercialization, 2) greater physician or patient demand for benznidazole stimulated by FDA approval, 3) efforts to increase awareness and blood donor screening that started even before commercialization, and 4) eligibility for treatment of patients who were not eligible for treatment under the CDC’s protocol for compassionate use. Interpretation of the increased use of benznidazole requires careful consideration.

It is important to note that our study did not find that drug price was a key access barrier for benznidazole, contrary to the concerns of some commentators who warned about access problems if the manufacturer set an excessively high price [29]. Access to benznidazole is facilitated by the company’s low-cost/free drug program, although affordability problems may still occur due to the costs of other medical services for uninsured and underinsured patients. Our study suggests that focusing only on drug price is not an adequate way to analyze access to medicine for benznidazole.

This analysis shows that many challenges remain in efforts to expand access to benznidazole in the US. We suggest six areas of strategic action to expand access to benznidazole, with a focus on the roles of Exeltis USA and FMS. These proposed actions are presented in order of relative importance considering the likely feasibility of implementation.

Area #1: Establish an emergency benznidazole delivery system. The CDC has requested that Exeltis USA develop an emergency delivery system for the small number of unusual cases that require benznidazole urgently. Exeltis USA is exploring solutions with Foundation Care. The company should develop an effective emergency service with sufficient and sustainable financing.

Area #2: Ensure the financial sustainability of the FastAccess Program. The financial sustainability of the FastAccess Program is critical to keep benznidazole affordable for patients without adequate insurance coverage. Exeltis USA and FMS should assess the program’s future financial costs and develop a plan to ensure sustainability. At the same time, Exeltis USA should continue expanding private and public insurance coverage of benznidazole to reduce reliance on the FastAccess program.

Area #3: Organize and educate treating providers. Education of treating providers is crucial to ensure and increase appropriate use of benznidazole. Exeltis USA could undertake changes in its benznidazole website: 1) increase visibility and clarity of the benznidazole order form to ensure that providers can easily satisfy paperwork requirements for ordering benznidazole, 2) develop and upload a self-assessment tool to help providers check their decisions on patient eligibility for treatment, and 3) add an online hub function where providers who are new to CD treatment can obtain advice from experienced providers. Exeltis USA could also collaborate with researchers and clinicians to investigate the extent to which patients are appropriately diagnosed and evaluated to establish treatment eligibility and use this information to improve educational resources for treating providers.

Area #4: Promote development of treatment guidelines. Clinical practice guidelines for CD treatment can help persuade more providers and hospitals to provide appropriate care for infected persons. FMS could support the establishment of a group of experts to write treatment guidelines and obtain endorsement by key medical societies.

Area #5: Promote patient peer support. Exchange of information and experiences among patients and those seeking care will help improve patient acceptance of treatment. Exeltis USA could collaborate with existing treatment centers to develop local patient networks. Exeltis USA could also create an online hub function in the website where care seekers can virtually interact with treated CD patients.

Area #6: Expand the FDA-approved indication for benznidazole. FDA approval of benznidazole for use in older children and adults would improve physician acceptance, although the drug is currently used mostly for treatment of adult patients. The efficacy of a short course of benznidazole in clearing *T*. *cruzi* parasitemia in adult patients was demonstrated recently in a phase II clinical trial in Bolivia [30], which needs to be confirmed by a phase III trial. FMS can consider investing in additional clinical studies and advancing scientific evidence, while Exeltis USA can apply to the FDA for an expanded indication for benznidazole.

In addition, we propose expanding the existing informal network, the Chagas Consortium, to strengthen coordination among different actors concerned with Chagas disease in the US. The expanded network could include not only researchers and clinicians but also government agencies and Exeltis USA. Coordination of different interests among a broader group of actors will facilitate strategic actions.

US government institutions could also play an important role to improve access to CD treatment. First, the CD experts at CDC and other government agencies could maintain a dialogue with Exeltis USA to push the government agenda for ensuring availability of safe and effective drugs, including establishment of the emergency benznidazole delivery system. Second, in order to strengthen disease surveillance, CD can be added to the list of reportable diseases by state governments as well as CDC’s national notifiable disease surveillance system. Surveillance efforts can help increase awareness among providers and assist in linking CD patients to treatment [31]. Third, the Office of Minority Health at US Department of Health and Human Services could develop policies to increase physician and patient awareness about CD among Hispanic/Latino American communities. Fourth, the Health Center Program at the Health Resources and Services Administration can create incentives or compliance manuals for community health centers to identify people infected with *T*. *cruzi*. In this program, community health workers would play a key role to provide logistical or emotional support to patients. Finally, a national health action plan for CD could be developed, as is the case with the other infectious diseases [32].

We recognize that access to CD diagnosis is also problematic in the US [10]. While this topic was not within the scope of this study, creating access to benznidazole will require actions in relation to diagnosis, such as assessment of the four FDA-cleared diagnostic assays [18,33], development of strategies for maternal and newborn screening, education of primary care providers on diagnostic testing, and improvement of linkage to care for seropositive blood donors. Likewise, we did not consider access to nifurtimox, which is still provided by CDC for compassionate use. An expanded Chagas Consortium could invite blood banks, companies in the diagnostic market and other pharmaceutical companies to join in efforts to expand access to CD diagnosis and treatment.

This study involves three key limitations. First, we did not necessarily identify and interview all actors who may have had a problem in providing or receiving CD treatment. In particular, we did not conduct interviews with CD patients. While we tried to capture patient views from interviews with health care providers and the literature [27], closer examination of patient experiences, particularly of those who are currently struggling to access CD diagnosis and treatment, may reveal different access barriers that this study was unable to identify. Second, our study intentionally focused on access to benznidazole, without considering broader problems with access to health care in the US. We recognize that access to a specific health product is situated within the greater challenges of the health system. By choosing a narrow definition of access, this study does not fully examine these broader considerations that exist for CD patients in the US. Third, we applied the access framework and used published and interview data to identify and assess factors that influence access to benznidazole and to propose interventions that can improve access. The relationships between those factors and access to benznidazole, however, involve some uncertainty, and additional research is needed to examine the implementation of the proposed actions and their consequences.

The findings and recommendations of this study are unique to the situation of benznidazole in the US and are not directly applicable to other countries or other products. However, the approach used in this study, particularly the explicit application of the Frost and Reich access framework to propose actions, could be useful in other cases, such as for developing access strategies for benznidazole in other countries or for other FDA-approved drugs for neglected diseases. While the literature analyzing access to PRV-granted drugs [14,29] focuses primarily on availability and affordability, our study also explored architecture, acceptability and appropriate use as important access components, providing a more comprehensive picture about access.

In conclusion, access to benznidazole continues to be limited in the US even after FDA approval and commercialization due to barriers identified in this study. We believe that the strategic actions proposed above are feasible, can help address the access barriers for benznidazole, and will contribute to expanding access to benznidazole in the US. In addition, the approach used in this study can be applied for designing access programs for benznidazole in other countries or for other health products in the US and elsewhere.
